# Sleep Disorders Reduce Health-Related Quality of Life in Multiple Sclerosis (Nottingham Health Profile Data in Patients with Multiple Sclerosis)

**DOI:** 10.3390/ijms160716514

**Published:** 2015-07-21

**Authors:** Christian Veauthier, Gunnar Gaede, Helena Radbruch, Klaus-Dieter Wernecke, Friedemann Paul

**Affiliations:** 1 Interdisciplinary Center for Sleep Medicine, Charité University Medicine Berlin, Charitéplatz 1, 10117 Berlin, Germany; 2 NeuroCure Clinical Research Center, Charité University Medicine Berlin, Charitéplatz 1, 10117 Berlin, Germany; E-Mails: gunnar.gaede@charite.de (G.G.); helena.radbruch@charite.de (H.R.); friedemann.paul@charite.de (F.P.); 3 Department of Neurology, St. Joseph Hospital Berlin-Weissensee, 13088 Berlin, Germany; 4 CRO SOSTANA GmbH and Charité University Medicine Berlin, Wildensteiner Straße 27, 10318 Berlin, Germany; E-Mail: wernecke@sostana.de; 5 Clinical and Experimental Multiple Sclerosis Research Center, Department of Neurology, Charité University Medicine Berlin, 10117 Berlin, Germany

**Keywords:** restless legs syndrome, sleep disorders, multiple sclerosis, clinical neurophysiology, polysomnography, insomnia, pain, depression, health, quality of life

## Abstract

Quality of Life (QoL) is decreased in multiple sclerosis (MS), but studies about the impact of sleep disorders (SD) on health-related quality of Life (HRQoL) are lacking. From our original cohort, a cross-sectional polysomnographic (PSG) study in consecutive MS patients, we retrospectively analysed the previously unpublished data of the Nottingham Health Profile (NHP). Those MS patients suffering from sleep disorders (*n* = 49) showed significantly lower HRQoL compared to MS patients without sleep disorders (*n* = 17). Subsequently, we classified the patients into four subgroups: insomnia (*n* = 17), restless-legs syndrome, periodic limb movement disorder and SD due to leg pain (*n* = 24), obstructive sleep apnea (*n* = 8) and patients without sleep disorder (*n* = 17). OSA and insomnia patients showed significantly higher NHP values and decreased HRQoL not only for the sleep subscale but also for the “energy” and “emotional” area of the NHP. In addition, OSA patients also showed increased NHP values in the “physical abilities” area. Interestingly, we did not find a correlation between the objective PSG parameters and the subjective sleep items of the NHP. However, this study demonstrates that sleep disorders can reduce HRQoL in MS patients and should be considered as an important confounder in all studies investigating HRQoL in MS.

## 1. Introduction

Recently, we published the results of a cross-sectional polysomnographic (PSG) study in consecutive multiple sclerosis (MS) patients [1]. Of 66 patients who underwent PSG, 49 suffered from a sleep disorder (SD); seven of these suffered from more than one SD. In these cases we classified only the more severe SD. In our study, SDs were significantly related to fatigue; and a follow-up investigation showed that a consequent treatment of sleep disorders may improve fatigue in a subset of patients [2]. The improvement of MS fatigue after medical treatment of SD was seen in another follow up study as well [3]. With regards to the relationship between health related Quality of Life (HRQoL) and SD in MS patients, there are only a few studies: Neau *et al.* [4], as well as Sarraf *et al.* [5], classified MS patients into good sleepers and poor sleepers using the Pittsburgh Sleep Quality Index [6] (PSQI) (PSQI ≤ 5 *vs.* > 5). In their studies, poor sleepers showed a reduced HRQoL using the MS-QOL-54 [7]. To our knowledge, there is only one study investigating the relationship between SDs confirmed by PSG and HRQoL in MS: Trojan *et al.* demonstrated a decreased mental but not physical HRQoL in MS patients with SD [8] using the Short Form Health Survey (SF-36) [9].

To date, there is no study investigating the HRQoL in MS patients with the Nottingham Health Profile (NHP) [10]. The NHP is a valid and reliable indicator of subjective health status in physical, social and emotional areas [10]. The NHP consists of two parts (part 1 and 2). Only part 1 is weighted and is composed of six subscales (*sleep*, *physical mobility*, *energy*, *pain*, *emotional reactions* and *social isolation*); the maximum of any subscale is 100. As a result, the maximum of the NHP total score is 600 (the higher the NHP values, the lower the HRQoL). The weighting of the 38 statements reflects the symptom severity and represents rather severe problems in order to avoid picking up a large number of false positives [10]. In the literature mine rescue workers show a very low global mean NHP score of 8.8; fit elderly persons show a mean global NHP score of 12.4; whereas pregnant women at 37 weeks, fracture victims and chronically ill elderly patients (mean global NHP 127.0/129.6/156.4) show increased NHP values, and especially high values were obtained in patients with osteoarthrosis (mean global NHP 271.3) [10]. Verwimp *et al.* investigated 75 OSA patients [11] and found an increased global NHP median (218). In their study the negative perception in the “physical abilities” domain was effectively related to an objective low level of physical activity measured by actigraphy.

In our previous cross-sectional trial we also collected NHP data, which had not been analysed and published before. The aim of this study is to describe these data and to investigate the relationship between SD and HRQoL in MS.

## 2. Results

### 2.1. Patients

We classified the 66 patients (21 men and 45 women aged 20–66 years) into four subgroups: no sleep disorder (NSD) (*n* = 17), insomnia (*n* = 17) (INS), periodic limb movement disorder (PLMD), restless legs syndrome (RLS) or SD due to leg pain (PLMD/RLS) (*n* = 24), and untreated obstructive sleep apnea (OSA) (*n* = 8). Expanded Disability Status Scale (EDSS) [12] values ranged from zero to eight.

#### 2.1.1. HRQoL in MS Patients with Sleep Disorders Compared to Patients without Sleep Disorders

Table 1 shows the NHP values in patients without SD compared with those patients suffering from SD (all SD together). MS patients suffering from SD showed significantly increased NHP values, indicating poorer HRQoL using the Mann–Whitney-*U*-test.

**Table 1:** NHP values in patients with and without sleep disorders.

NHP Global Score and Subscales	Average and Range	All Patients	Patients without Sleep Disorders	Patients with Sleep Disorders	Differences between the Two Subgroups
NHP-Total	Mean (±standard deviation)	146.1 (±119.8)	67.3 (±60.0)	175.2 (±123.6)	*p* = 0.001
	Min–Max	0.0–78.7	0.0–188.6	0.0–413.7	
	25–75	0.0–32.6	21.8–120.5	61.3–273.3	
	median	126.4	34.8	175.5	
Physical abilities	Mean (±standard deviation)	20.9 (±21.5)	10.2 (±14.7)	24.8 (±22.4)	*p* = 0.010
	Min–Max	0.0–78.7	0.0–54.5	0.0–78.7	
	25–75	0.0–32.6	0.0–22.0	10.8–36.5	
	median	12.7	0.0	21.7	
Social isolation	Mean (±standard deviation)	11.7 (±19.6)	3.6 (±8.0)	14.7 (±21.8)	*p* = 0.048
	Min–Max	0.0–80.6	0.0–22.5	0.0–80.6	
	25–75	0.0–20.1	0.0–0.0	0.0–22.5	
	median	0.0	0.0	0.0	
Sleep	Mean (±standard deviation)	29.3 (±29.5)	10.6 (±15.1)	36.2 (±30.6)	*p* = 0.001
	Min–Max	0.0–100.0	0.0–50.4	0.0–100.0	
	25–75	0.0–16.1	0.0–12.6	12.6–72.7	
	median	50.4	0.0	28.7	
Pain	Mean (±standard deviation)	15.8 (±24.8)	3.8 (±9.8)	20.2 (±27.2)	*p* = 0.009
	Min–Max	0.0–100.0	0.0–32.3	0.0–100.0	
	25–75	0.0–0.0	0.0–0.0	0.0–30.6	
	median	26.0	0.0	9.9	
Energy	Mean (±standard deviation)	48.9 (±40.9)	29.9 (±35.5)	55.8 (±40.9)	*p* = 0.016
	Min–Max	0.0–100.0	0.0–100.0	0.0–100.0	
	25–75	0.0–60.8	0.0–62.0	24.0–100.0	
	median	100.0	0.0	62.0	
Emotional	Mean (±standard deviation)	19.6 (±18.8)	9.3 (±12.2)	23.5 (±19.5)	*p* = 0.006
	Min–Max	0.0–69.0	0.0–41.4	0.0–69.0	
	25–75	0.0–30.9	0.0–18.6	5.3–41.4	
	median	16.8	0.0	21.0	

#### 2.1.2. Comparison of the Global NHP Values (Global HRQoL) in the Four Subgroups

**Figure 1:**
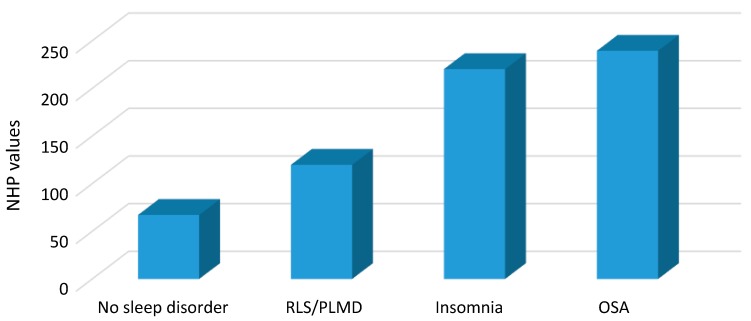
Nottingham Health Profile total values in the four subgroups.

The comparison of the global NHP (including all six subscales) in the four subgroups showed significantly lower NHP values in the NSD and PLMD/RLS patients compared to OSA and insomnia patients; whereas there were no significant differences between NSD and PLMD/RLS patients neither between OSA and insomnia patients (see Figure 1 and Table 2). This suggests that NSD and PLMD/RLS patients have a better global HRQoL compared to OSA or insomnia patients.

**Table 2:** NHP total score and NHP items in the different subgroups.

NHP Values	Average and Range	All Patients	NSD	INS	OSA	PLM	Differences between the Two Subgroups
Total	Mean (SD)	146.1	67.3	220.3	239.6	119.9	NSD *vs.* OSA *p* = 0.003 NSD *vs.* INS *p* < 0.0001 INS *vs.* PLM *p* = 0.002 OSA *vs.* PLM *p* = 0.042 NSD *vs.* PLM *p* = 0.210 OSA *vs.* INS *p* = 0.804
	Standard deviation	119.8	60.0	88.2	136.2	123.7	
	Min–Max	0.0–78.7	0.0–188.6	60.7–369.9	24.7–413.7	0.0–408.61	
	25–75	0.0–32.6	21.8–120.5	147.0–276.0	180.8–393.3	29.7–174.4	
	Median	126.4	34.8	212.3	194.9	75.8	

#### 2.1.3. Comparison of the NHP Subscales in the Four Subgroups

**Figure 2:**
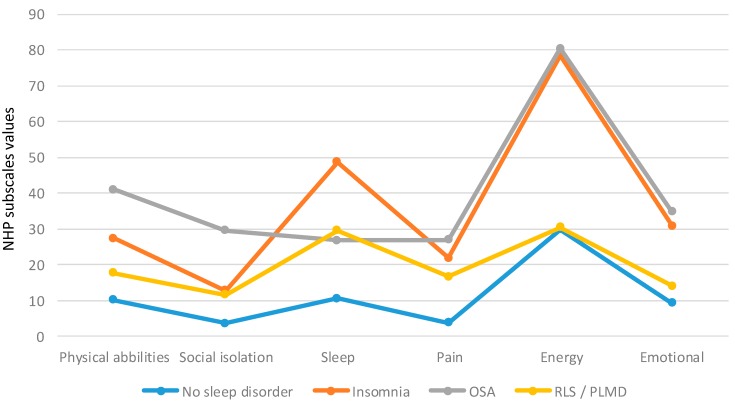
Subscales of the NHP in the different sleep disorders (mean values).

Figure 2 displays the NHP values in the different subgroups. The patients without sleep disorders showed the lowest NHP values in all items. The insomnia subgroup showed the highest values in the “*sleep*” item. Attention should also be paid to the high values concerning “*energy*” (and to a lower extent regarding “*emotions*”) in the insomnia and OSA subgroup. Please take into account the high values regarding “*physical abilities*” in the OSA subgroup.

#### Kruskal–Wallis-Test

We performed the non-parametric Kruskal–Wallis-Test for comparing the four subgroups with different sample sizes. The Kruskal–Wallis-Test showed significant differences between the four subgroups for all items except for “*social isolation*”, meaning that this item seemed not to be different in the four subgroups—therefore, this item was not included in the further analysis (see Table 3).

**Table 3:** Kruskal–Wallis-Test.

NHP Total	Physical Abilities	Social Isolation	Sleep	Pain	Energy	Emotional
*p* < 0.0001	*p* = 0.007	*p* = 0.054	*p* = 0.001	*p* = 0.034	*p* < 0.0001	*p* < 0.0001

#### Mann–Whitney-*U*-Test

We subsequently analysed the NHP subscales (except for “*social isolation*”): a Mann–Whitney-*U*-test was performed in order to analyse the differences between two specific subgroups (OSA–PLM/OSA–INS/OSA–NSD/PLM–INS/PLM–NSD/INS–NSD). Five items (*physical abilities*, *sleep*, *pain*, *energy*, *emotional*) remained in the further analysis (see Table 3).

In four subscales (*physical abilities*, *sleep*, *energy*, *emotional*) we found significantly lower NHP values in NSD patients compared to insomnia and OSA patients (in the *pain* area there was only a significant difference between NSD patients and insomnia patients—but not between NSD patients and OSA patients) (see Table 4). This means that the HRQoL in these specific areas was higher in MS patients without comorbid sleep disorders compared to MS patients suffering from comorbid OSA or insomnia.

**Table 4:** NHP subscale values in all patients and in the four subgroups.

Subscales	Average and Range	All Patients	NSD	INS	OSA	PLM	Differences between the Two Subgroups
Physical abilities	Mean (Standard deviation)	20.9 (21.5)	10.2 (14.7)	27.4 (20.8)	41.0 (24.3)	17.7 (20.8)	NSD *vs.* OSA *p* = 0.003 NSD *vs.* INS *p =* 0.009 INS *vs.* PLM *p* = 0.138 OSA *vs.* PLM *p =* 0.032 NSD *vs.* PLM *p =* 0.211 OSA *vs.* INS *p =* 0.260
	Min–Max	0.0–78.7	0.0–54.5	0.0–77.3	10.8–78.2	0.0–78.7	
	25–75	0.0–32.6	0.0–21.9	10.8–42.6	21.7–67.2	0.0–25.8	
	Median	12.7	0.0	22.0	32.6	11.2	
Social isolation	Mean (Standard deviation)	11.7 (19.6)	3.6 (8.0)	12.8 (19.0)	29.5 (31.0)	11.6 (19.4)	For this subgroup no Mann–Whitney-*U*-Test was performed (see Table 2)
	Min–Max	0.0–80.6	0.0–22.5	0.0–64.7	0.0–80.6	0.0–63.9	
	25–75	0.0–20.1	0.0–0.0	0.0–22.5	0.0–63.9	0.0–20.2	
	Median	0.0	0.0	0.0	20.1	0.0	
Sleep	Mean (Standard deviation)	29.3 (29.5)	10.6 (15.2)	48.7 (26.7)	26.8 (27.4)	29.5 (32.4)	NSD *vs.* OSA *p =* 0.087 NSD *vs.* INS *p* < 0.0001 INS *vs.* PLM *p* = 0.048 OSA *vs.* PLM *p* = 0.980 NSD *vs.* PLM *p* = 0.063 OSA *vs.* INS *p* = 0.075
	Min–Max	0.0–100.0	0.0–50.4	0.0–77.6	0.0–77.6	0.0–100	
	25–75	0.0–16.1	0.0–12.6	25.2–75.2	12.6–50.4	0.0–50.4	
	Median	50.4	0.0	50.4	12.6	14.3	
Pain	Mean (Standard deviation)	15.8 (24.8)	3.8 (9.8)	21.9 (23.9)	27.0 (31.4)	16.6 (28.9)	NSD *vs.* OSA *p* = 0.114 NSD *vs.* INS *p =* 0.012 INS *vs.* PLM *p* = 0.221 OSA *vs.* PLM *p* = 0.469 NSD *vs.* PLM *p =* 0.117 OSA *vs.* INS *p =* 0.804
	Min–Max	0.0–100.0	0.0–32.7	0.0–69.8	0.0–80.2	0.0–100.0	
	25–75	0.0–0.0	0.0–0.0	0.0–40.1	0.0–56.9	0.0–18.6	
	Median	26.0	0.0	15.8	26.0	0.0	
Energy	Mean (Standard deviation)	48.9 (40.9)	29.9 (35.5)	78.5 (28.7)	80.5 (38.0)	30.5 (35.4)	NSD *vs.* OSA *p* = 0.007 NSD *vs.* INS *p* < 0.0001 INS *vs.* PLM *p* < 0.0001 OSA *vs.* PLM *p* = 0.013 NSD *vs.* PLM *p* = 0.790 OSA *vs.* INS *p* = 0.710
	Min–Max	0.0–100.0	0.0–100.0	24.0–100.0	0.0–100.0	0.0–100.0	
	25–75	0.0–60.8	0.0–62.0	60.8–100.0	63.2–100.0	0.0–61.4	
	Median	100.0	0.0	100.0	100.0	24.0	
Emotional	Mean (Standard deviation)	19.6 (18.8)	9.3 (12.2)	30.9 (19.8)	34.8 (16.8)	14.1 (15.9)	NSD *vs.* OSA *p* = 0.001 NSD *vs.* INS *p* = 0.001 INS *vs.* PLM *p* = 0.007 OSA *vs.* PLM *p* = 0.008 NSD *vs.* PLM *p* = 0.392 OSA *vs.* INS *p* = 0.619
	Min–Max	0.0–69.0	0.0–41.4	0.0–69.0	13.6–55.9	0.0–48.5	
	25–75	0.0–30.9	0.0–18.6	14.1–47.1	17.0–52.0	0.0–22.7	
	Median	16.8	0.0	30.9	30.9	10.9	

In sum, the differences between the OSA and insomnia subgroups were very small and not significant. Similarly, the differences between NSD and the PLMD/RLS patients were negligible. Significant clinical relevant differences were found comparing NSD and the PLMD/RLS patients to OSA and insomnia patients.

The comparison between PLMD/RLS patients and insomnia patients showed significantly increased NHP values in the “sleep” subscale and highly significant increased NHP values in the “*energy*” and “*emotional*” subscale. That means that insomnia patients showed a reduced HRQoL in these areas compared to PLMD/RLS patients.

When comparing PLMD/RLS patients with OSA patients, there were significantly higher NHP values in OSA patients (decreased HRQoL) in the “*physical abilities*”, “*energy*” and “*emotional*” subscales.

#### 2.1.4. Comparison of the Objective (PSG) Sleep Parameters and the NHP Sleep Items

The sleep subscales consist of five items: “I sleep badly at night”, “I lie awake for most of the night”, “It takes me a long time to get to sleep”, “I’m waking up in the early hours of the morning”, “I take pills to help me sleep”. Except for the last item (“I take pills to help me sleep”), we compared the other four items with PSG parameters using the Mann–Whitney-*U*-test: Table 5 shows the results:

**Table 5:** Comparison of polysomnographic data and NHP sleep items.

Items	Average and Range	Sleep Efficiency	Awakenings	Arousal-Index	Sleep Latency	Wake after Sleep Onset
I sleep badly at night ***YES***	Mean (±standard deviation)	73.6 (±12.6)	25.5 (±7.7)	18.5 (±9.6)		
	Min–Max	50–94	9–41	3.9–43.9		
	25–75	63.7–83.5	20–30.5	12.2–22.9		
	median	74.8	26.0	16.5		
I sleep badly at night ***NO***	Mean (±standard deviation)	76.7 (±16.2)	27.5 (±14.2)	20.5 (±10.0)		
	Min–Max	8–93	8–72	1.1–47.1		
	25–75	73.0–87.1	17.8–33.3	14.4–24.9		
	median	80.45	26.0	21.7		
Differences between ***YES*** and ***NO***		*p* = 0.148	*p* = 0.860	*p* = 0.255		
I lie awake for most of the night ***YES***	Mean (±standard deviation)	76.2 (±15.0)				
	Min–Max	8–93				
	25–75	69.8–87.0				
	median	79.7				
I lie awake for most of the night ***NO***	Mean (±standard deviation)	71.1 (±13.5)				
	Min–Max	50–94				
	25–75	60.0–80.7				
	median	69.6				
Differences between ***YES*** and ***NO***		*p* = 0.175				
It takes me a long time to get to sleep ***YES***	Mean (±standard deviation)				38.5 (±39.8)	
	Min–Max				2–198	
	25–75				15.3–49.5	
	median				29.0	
It takes me a long time to get to sleep ***NO***	Mean (±standard deviation)				26.4 (±31.1)	
	Min–Max				0–190	
	25–75				11.0–32.0	
	median				21.0	
Differences between ***YES*** and ***NO***					*p* = 0.08	
I’m waking up in the early hours of the morning ***YES***	Mean (±standard deviation)					88.4 (±59.0)
	Min–Max					27–258
	25–75					43.0–73.0
	median					73.0
I’m waking up in the early hours of the morning ***NO***	Mean (±standard deviation)					69.8 (±41.4)
	Min–Max					20–173
	25–75					43.3–88.8
	median					52.5
Differences between ***YES*** and ***NO***						*p* = 0.336

When we compared “*It takes me a long time to get to sleep*” to the sleep latency measured by PSG, there was no significant correlation between this subjective (NHP) and objective (PSG) measurement of sleep latency. Furthermore, we did not find any correlation between “*I sleep badly at night*” and sleep efficiency measured by PSG. Similarly the item “*I’m waking up in the early hours of the morning*” did not correlate with wake-after-sleep-onset in the PSG nor arousal-index or awakenings.

### 2.2. Correlation between NHP Values and Other Questionnaires

Table 6 shows the non-parametric correlations (Spearman–Rho) between NHP and other self-assessed questionnaires (Modified Fatigue Impact Scale (MFIS) [13]; Beck Depression Inventory (BDI) [14]; Pittsburgh Sleep Quality Index (PSQI) [6]).

**Table 6:** Non parametric correlations (Spearman-Rho) between NHP and other questionnaires (Beck Depression Inventory and Pittsburgh Sleep Quality Index).

NHP and MFIS	NHP and BDI	NHP and PSQI
*p* < 0.0001	*p* < 0.0001	*p* < 0.0001
*r* = 0.737	*r* = 0.836	*r* = 0.612

**Figure 3:**
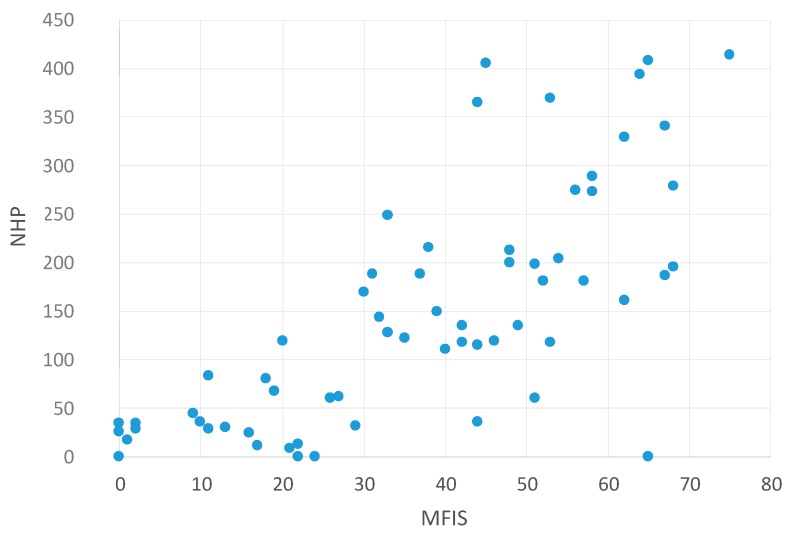
Correlation between NHP and MFIS values. Abbreviations: NHP = Nottingham Health Profile; MFIS = Modified Fatigue Impact Scale [13].

**Figure 4:**
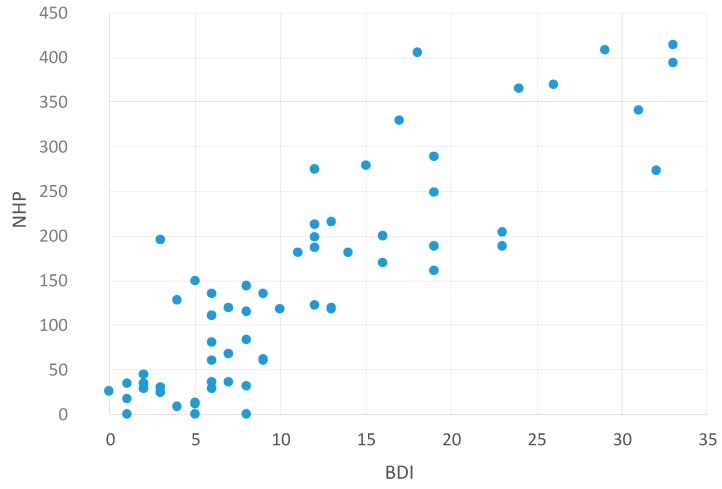
Correlation between NHP and BDI values. Abbreviations: NHP = Nottingham Health Profile; BDI = Beck Depression Inventory [14].

**Figure 5:**
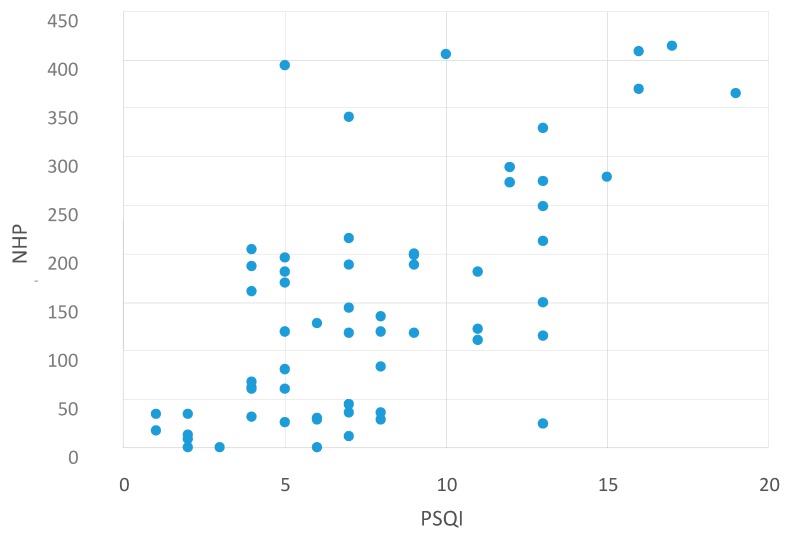
Correlation between NHP and PSQI values. Abbreviations: NHP = Nottingham Health Profile; PSQI = Pittsburgh Sleep Quality Index [6].

The scatter plots visualize these findings. There was a significant correlation between NHP values and MFIS values—meaning that higher fatigue values are associated with reduced HRQoL (Figure 3). In addition, higher NHP values (reduced HRQoL) were also associated with higher depression values (BDI, Figure 4) and higher PSQI values (low sleep quality, Figure 5). This indicates that reduced HRQoL is associated with depression, fatigue, and bad sleep quality.

#### Correlation between NHP Values and the MFIS Subscales

Furthermore, we investigated the correlation between the NHP global score and the three subscales of the MFIS (cognition, psychosocial and physical): a significant correlation was found between the global HRQoL (NHP values) and psychosocial aspects of fatigue, as well cognitive fatigue and physical fatigue (see Table 7):

**Table 7:** Non-parametric correlations (Spearman–Rho) between NHP and the fatigue subscales.

NHP and Cognitive MFIS-Subscale	NHP and Physical MFIS-Subscale	NHP and Psychosocial MFIS-Subscale
*p* < 0.0001	*p* < 0.0001	*p* < 0.0001
*r* = 0.635	*r* = 0.726	*r* = 0.548

Abbreviations: NHP = Nottingham Health Profile; MFIS = Modified Fatigue Impact Scale.

## 3. Discussion

Our study demonstrates poor HRQoL in MS patients suffering from sleep disorders confirmed by PSG—especially from OSA and insomnia. The global NHP median was marginally lower in MS patients suffering from OSA (194.9) compared with OSA patients in the general population (218.0) [11]. Increased NHP values indicate severe health problems, and the mean global NHP score in MS patients with comorbid insomnia or OSA was higher than the mean scores described in the literature in pregnant women at 37 weeks, fracture victims and chronically ill elderly patients and almost as high as in patients with osteoarthrosis, whereas MS patients without sleep disorders show only moderately increased NHP values. OSA and insomnia can significantly reduce HRQoL in MS patients. MS patients suffering from PLMD, RLS or sleep disorders due to leg pain show a decreased HRQoL as well—although to a lesser extent.

The impairment of HRQoL in OSA and insomnia patients (besides the sleep problems) was more pronounced in the “*energy*” and “*emotional*” area. OSA patients are severely affected in the “*physical abilities*” as well (as described previously by Verwimp *et al.* in OSA patients without MS [11]).

We cannot explain the lack of a correlation between the (objective) PSG parameter and the (subjective) sleep problems measured in the sleep subscale. This could be due to the fact that we investigated this relationship in different SDs (OSA, insomnia, PLMD/RLS).

The decreased HRQoL in OSA and insomnia patients in the “*energy*” and “*emotional*” area argue for consecutive daytime symptoms due to the sleep disorders (and “*physical abilities*” in OSA patients as well). Here, it is difficult to explain what exactly drives these daytime symptoms. As recently reported [15], there is an overlap between fatigue, pain, depression, and sleep disorders. Moreover, OSA can lead to depression and continuous positive airway (CPAP) therapy can improve depression [16]. Insomnia has been found to be a clinical predictor of subsequent depression [17] and increased PSQI values are significantly associated with fatigue in MS patients [18]. To date, evidence-based therapies of MS-related fatigue are lacking [19,20], and patients without MS suffering from sleep disorders show equally high values on the fatigue scales (MFIS and FSS) [21].

Sleep disorders can lead to fatigue [21] and depression [16] and CPAP therapy can subsequently improve these symptoms in patients with sleep apnea [15,22]. This suggests that reduced HRQoL, fatigue and depression can be common features of sleep disorders. Due to the close and complex relationship between fatigue, depression and sleep disorders in MS, and the overlap of the used questionnaires [15], we cannot state if sleep disorders lead to depression and subsequently to decreased HRQoL in the “*energy*” and “*emotional*” area—or *vice versa*, if sleep disorders lead to reduced daytime functioning and, subsequently, to depression.

Our findings underscore that sleep disorders should be considered an important confounder in all future studies investigating HRQoL in MS patients.

## 4. Experimental Section

### 4.1. Literature Search

A literature search was performed until May 2015 in PubMed (http://www.ncbi.nlm.nih.gov/pubmed) with the following keywords: “multiple sclerosis AND Nottingham Health Profile” and “multiple sclerosis AND quality of life”. After reading the abstracts, only relevant articles were read. Moreover, the references of these articles were read and hand-searched for potentially relevant studies or articles as well.

### 4.2. Patients

We classified the 66 patients (21 men and 45 women aged 20–66 years) into four subgroups: no sleep disorder (NSD) (*n* = 17), insomnia (*n* = 17), periodic limb movement disorder (PLMD), restless legs syndrome (RLS) or SD due to leg pain (PLMD/RLS) (*n* = 24), and untreated obstructive sleep apnea (OSA) (*n* = 8).

Expanded Disability Status Scale (EDSS) [12] values ranged from zero to eight. For more demographic details please see the original article [1]. All patients completed the NHP [10], MFIS [13], BDI [14], and the PSQI [6] in a German validation [23,24,25,26].

The original study was approved by the local ethics committees (Charité University Medicine Berlin, Berlin, Germany and Ernst Moritz Arndt University Medicine, Greifwald, Germany, project identification code BB 03/08; 31 January 2008), and all participants gave written informed consent prior to the assessment.

### 4.3. Data Collection

Data collection and extraction from the questionnaires (NHP) was performed by the corresponding author (CV). The PSG data extraction from the original study and the extraction of all questionnaires were performed by the corresponding author as well [1].

### 4.4. Polysomnography and Scoring Criteria

As described in our original article [1], we performed PSG using a mobile polysomnographic device worn on the body, which has been validated in three different sleep centers [27] (Somnocheck 2R&K, Weinmann Medical Technology; software: Somnolab; analysis software: Artisana, Hamburg, Germany) without a video or audio signal, but otherwise with full recording facilities as in a sleep laboratory.

Measurements were made over a period of 8 h: C3/C4-EEG electrodes to the contralateral mastoid electrode, ground electrode, electrooculogram on the ipsilateral mastoid electrode, bipolar chin electromyogram (EMG) of the muscle mentalis or muscle submentalis (according to biosignals testing and anatomical conditions), nasal airflow, thoracic breathing, abdominal breathing, position sensor, snoring signal, pulse oxymetry, pulse, electrocardiogram, bipolar 2-point EMG electrodes on both anterior tibial muscles. Prior to each measurement, an impedance test and a biosignal test were performed. A sleep specialist who was blinded to the clinical situation and the questionnaires analysed PSGs. Visual classification of sleep stages took place manually in accordance with Rechtschaffen and Kales [28]. Respiratory events were manually classified using the diagnostic guidelines of the Task Force of the American Academy of Sleep Medicine [29]. Periodic leg movements were pre-classified by the equipment’s software and manually corrected using the Coleman criteria [30]. We also investigated the hypnogram: sleep efficiency, sleep onset latency, sleep stages, wake-time after sleep onset, number of waking events, number of changes in sleep stages, arousal index, periodic leg movement (PLM) index, PLM index in rapid-eye-movement (REM) sleep and non-REM sleep, PLM arousal index in REM sleep and non-REM sleep, respiratory disturbance index (RDI), blood oxygen desaturation, as well as chin EMG tonus, all respiratory events depending on position, arousal and sleep stage, and further standard polysomnographic parameters. Due to the first-night effect (patient is not yet familiar with the polysomnographic device), no pathological findings were assessed from the first-night hypnogram. On the first night, only PLMs and respiratory and cardiac events were considered. Following classification of the PSGs, sleep histories were obtained (CV), and a sleep diagnosis was made according to the International Classification of Sleep Disorders second edition (ICSD-2) [31]. To avoid false conclusions with respect to mild sleep disorders as possible causes of tiredness, mild insomnias, nocturia, mild PLMDs and sleep-related breathing disorders with RDI below 10 per hour were not considered relevant sleep disorders. We classified as relevant sleep disorders only sleep disorders with disturbed hypnogram, which are able to cause consecutive daytime sleepiness.

### 4.5. Statistical Analyses

The results were expressed as mean, standard deviation, and range. Patients were classified into four subgroups by the presence of a sleep disorder. Following an exploratory analysis of the data the non-parametric Kruskal–Wallis-Test and subsequently the Mann–Whitney-*U*-Test for pairwise comparisons were performed. Non-parametric correlations (Spearman–Rho) were carried out.

Statistical significance was established at *p* < 0.05. Due to the exploratory nature of the study, all tests were performed as exploratory data analyses, such that no adjustments for multiple testing have been made. Analysis was performed with SPSS software (IBM^©^ SPSS^©^ Statistics, Version 21, ^©^Copyright 1989, 2010 SPSS Inc. an IBM Company, Chicago, IL, USA).

## 5. Conclusions

Sleep disorders can decrease HRQoL in MS patients—especially in the “*energy*” and “*emotional*” areas. In OSA patients, the “*physical abilities*” area can be negatively impacted as well. Future studies should investigate the impact of the treatment of sleep disorders on HRQoL in MS patients.
